# Supplementary material for: Distribution, Vertical Transmission, and Cooperative Mechanisms of Obligate Symbiotic Bacteria in the Leafhopper Maiestas dorsalis (Hemiptera, Cicadellidea)
Source: Insects. 2023 Aug 14;14(8):710. doi: 10.3390/insects14080710 (PMC10455556; doi:10.3390/insects14080710)
Supplement: Supplementary file 1 [file insects-14-00710-s001.zip › Supplementary Figure.docx]

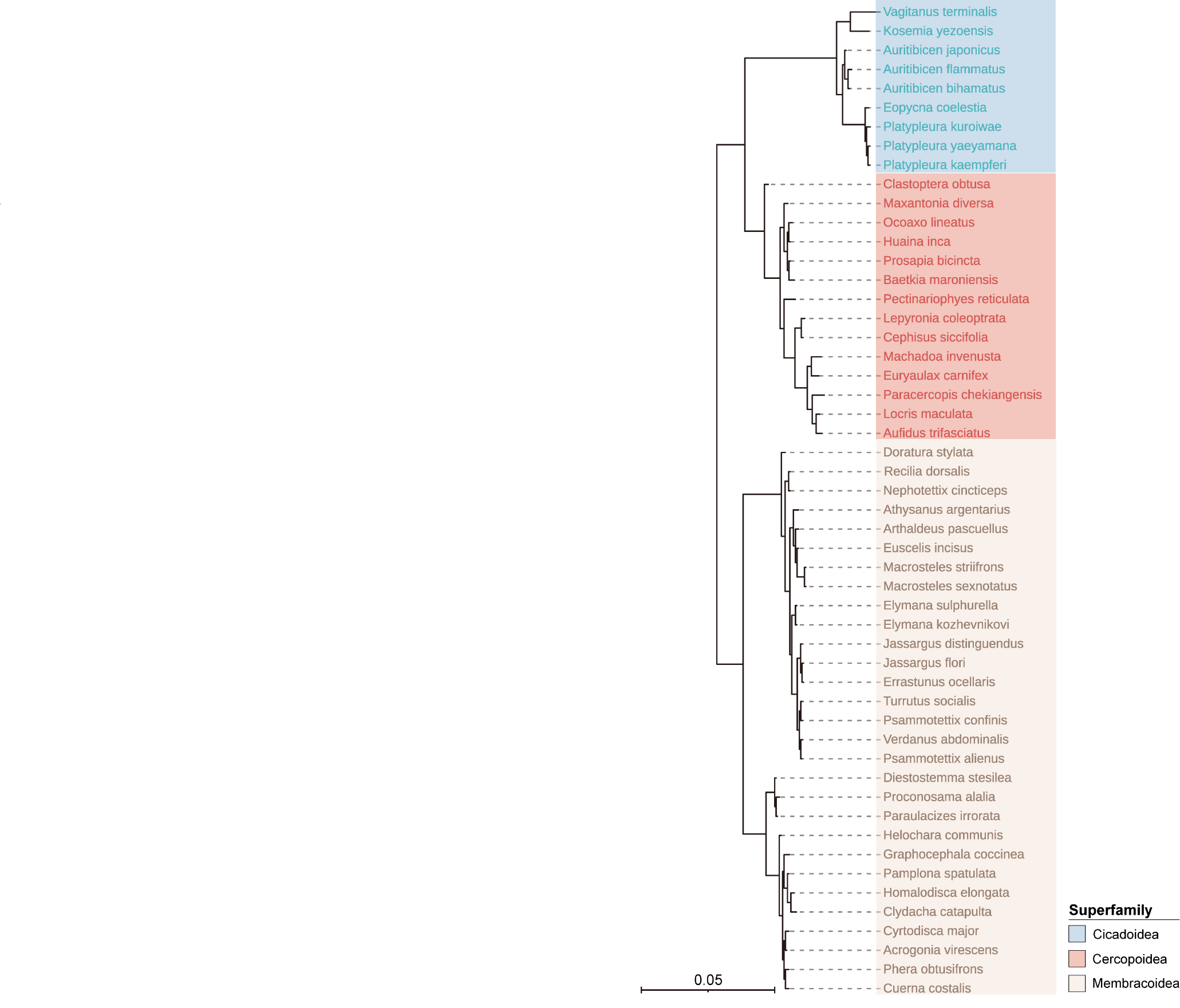


**Supplementary Figure 1. Maximum-likelihood phylogeny from reconstructed 16S rRNA genes.** We used IQ-Tree to construct a phylogenetic tree based on 16S rRNA sequences of *Sulcia* obtained from 53 Auchenorrhyncha insects. Terminals of *Sulcia* were labeled with the corresponding host taxon name.


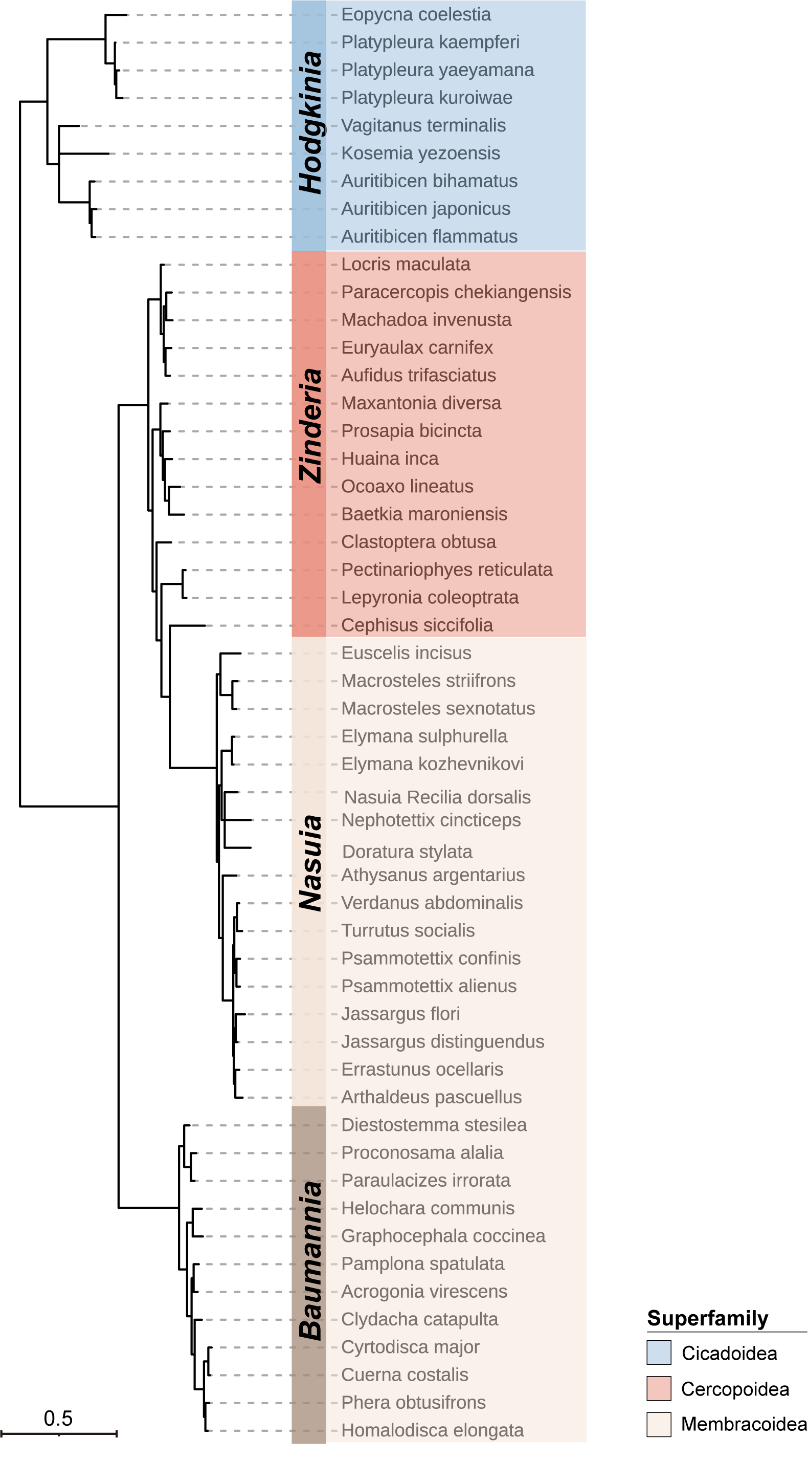


**Supplementary Figure 2. Maximum-likelihood phylogeny from reconstructed 16S rRNA genes.** We used IQ-Tree to construct a phylogenetic tree based on 16S rRNA sequences of *proteobacterial partners (including Nasuia, Baumannia, Zinderia, and Hodgkinia)* obtained from 53 Auchenorrhyncha insects. Terminals of *Sulcia* were labeled with the corresponding host taxon name. The proteobacterial partners were additionally labeled with the specific bacterial name on the left.


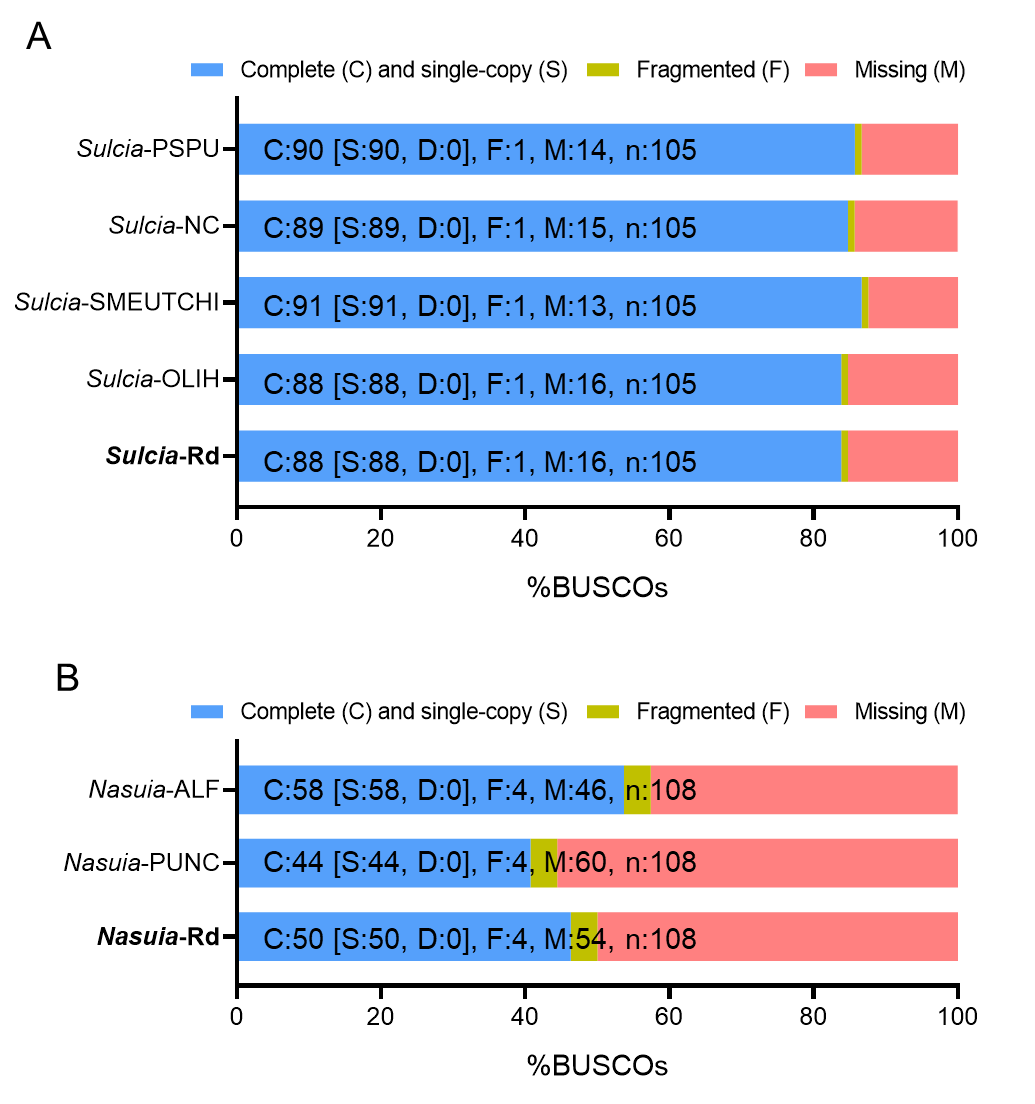


**Supplementary Figure 3. Assessment of genome completeness for *Sulcia* (A) and *Nasuia* (B).** Completeness was assessed with the BUSCO pipeline, which uses lineage-specific ortholog sets of conserved genes called BUSCOs. For each species, bars show the percent of BUSCOs from the reference lineage that were unambiguously found in the genome either as a single copy or duplicated, as well as those that were only partially found or were missing.
